# Sagittal Spinal Alignment in People with Chronic Spinal Cord Injury and Normal Individual: A Comparison Study Using 3D Ultrasound Imaging

**DOI:** 10.3390/jcm12113854

**Published:** 2023-06-05

**Authors:** Niraj Singh Tharu, Timothy Tin-Yan Lee, Kelly Ka-Lee Lai, Ting-Er Lau, Chui-Yi Chan, Yong-Ping Zheng

**Affiliations:** 1 Department of Biomedical Engineering, The Hong Kong Polytechnic University, Hong Kong SAR, China; 2 Research Institute for Smart Ageing, The Hong Kong Polytechnic University, Hong Kong SAR, China

**Keywords:** sagittal spinal alignment, thoracic kyphosis, lumbar lordosis, rehabilitation, spinal cord injury

## Abstract

The aim of this study was to compare the sagittal spinal alignment of people with chronic spinal cord injury (SCI) with normal individuals and to determine whether transcutaneous electrical spinal cord stimulation (TSCS) could cause a change in the thoracic kyphosis (TK) and lumbar lordosis (LL) to re-establish normal sagittal spinal alignment. A case series study was conducted, wherein twelve individuals with SCI and ten neurologically intact subjects were scanned using 3D ultrasonography. In addition, three people with SCI having complete tetraplegia participated further to receive a 12-week treatment (TSCS with task-specific rehabilitation) after evaluation of sagittal spinal profile. Pre- and post-assessments were conducted to evaluate the differences in sagittal spinal alignment. The results showed that the TK and LL values for a person with SCI in a dependent seated posture were greater than those of normal subjects for: standing (by TK: 6.8° ± 1.6°; LL: 21.2° ± 1.9°), sitting straight (by TK: 10.0° ± 4.0°; LL: 1.7° ± 2.6°), and relaxed sitting (by TK: 3.9° ± 0.3°; LL: 7.7° ± 1.4°), respectively, indicating an increased risk for spinal deformity. In addition, TK decreased by 10.3° ± 2.3° after the TSCS treatment, showing a reversible change. These results suggest that the TSCS treatment could be used to restore normal sagittal spinal alignment for individuals with chronic SCI.

## 1. Introduction

Spinal cord injury (SCI) is a disastrous situation resulting from neurological damage causing motor and sensory disturbances, leading to long-term disability [1,2]. People with SCI experience significant anatomical alignment changes [3], developing musculoskeletal disorders and impairments during their lifetimes [4]. Moreover, with progression in the chronic stage of SCI and due to muscular weakness, alignment of the spine is further interrupted (referred to as “malalignment”) [5,6], leading to progression of spinal deformities [7,8]. One of the most significant clinical problems reported is spinal imbalance [9,10]. It restricts the mobility and functions of the spine, affecting their daily life tasks [11,12]. In addition, they are at higher risk for fall-related injuries [10,13]. Therefore, the prevention of the progression of spinal deformities continues to be a critical aspect of the care for individuals with SCI [14].

The normal human spine in the sagittal alignment appears as “S-shaped” [15] and has lordotic curves in the cervical and lumbar regions with a kyphotic curve in thoracic region [16,17,18]. In addition, the normal spinal curvature has an impact on one’s body’s ability to perform daily tasks [14]. There are differences in the degrees of normal curvature, but these curves nonetheless enable the transfer of forces uniformly throughout the spinal column [16] and prevent excessive mobility [19].

Sagittal spinal alignment (SSA) refers to ideal and normal alignment of the spine in the sagittal plane [15]. The normal SSA is maintained by the ligaments and muscles that provide optimal strength and stability to the spine [20,21]. Additionally, it helps to establish the sagittal balance that allows one to keep the standing position without external support and with little muscle effort [20] and determines the quality of standing and sitting positions [22].

Disturbances of normal sagittal curvature could have direct and negative impacts on the general function and health-related quality of life of the individual [23]. In addition, the sagittal alignment of the spine in healthy individuals can alter due to various reasons such as degeneration, trauma, ageing, disease, and low back disorders [20] and could be exaggerated by poor posture [24]. Furthermore, an increment or decrement in the angle of spinal curvatures could result in spinal malalignment [25] that indicates an excess or deficit of normal lordosis or kyphosis [16]. Patients with kyphosis often report reduced walking abilities and impaired balance [26], while a hyperlordosis could increase the risk of facet joint arthritis [27].

Around 70–80 percent of people with SCI are confined to wheelchairs [28] and thus have extra difficulty to maintain a good posture [29]. The compensatory and abnormal postures are generally adopted by prolonged wheelchair users affected by SCI. This results in the development of a C-shaped spinal sagittal profile due to hyperkyphosis, hypolordosis, and posteriorly tilted pelvis [30,31]. Development of a C-shaped sagittal profile would increase the risk of spinal curvature disorders (SCD) in people with chronic SCI [31,32,33]. We believe that the abnormal posture obtained for a prolonged period in a wheelchair induces sagittal malalignment leading to SCDs. Therefore, preservation or restoration of neutral sagittal alignment remains a crucial issue for individuals with chronic SCI.

Treatments of SCDs include supportive care, bracing, and surgery, which have higher infection rates and higher treatment costs, while wearing braces for longer periods has been reported to be painful [34,35,36]. The proposed treatment in this study, transcutaneous electrical spinal cord stimulation (TSCS), is an emerging treatment modality for SCI rehabilitation [37,38] with demonstrated long-term treatment effects on trunk recovery [39]. It has also been reported that TSCS could reduce the uncomfortable feeling induced by wearing braces for people with SCDs [39]. Previous studies have demonstrated that using TSCS produced immediate trunk self-control in individuals with chronic SCI with a more steady and upright sitting position [13] and quickly restored the ability to sit up straight [40]. In addition, our recent study demonstrated that TSCS with task-specific rehabilitation (TSR) improved independent trunk control with increased static and dynamic sitting balance, developing a better erect upright sitting posture in individuals with complete chronic tetraplegia [39]. Therefore, we hypothesized that this intervention could be effective for restoring the normal sagittal alignment. The significance of this study was to compare the sagittal profile between people with chronic SCI and a normal individual using 3D ultrasound as well as to investigate how TSCS treatment corrects sagittal alignment.

The objectives of this study were to (1) compare the sagittal spinal profile between people with chronic SCI and normal individual in three different postures; and (2) determine the effectiveness of TSCS treatment in reducing thoracic kyphosis and lumbar lordosis for re-establishing normal sagittal alignment. The outcomes of this study would provide insight into the relationship between sagittal spinal alignment and progression of spinal sagittal deformity. In addition, this may assist in understanding the sagittal spinal profile in SCI, while the findings could benefit the prevention and management of spinal malalignment, providing a potential treatment for managing SCDs in individuals with chronic SCI.

## 2. Methods

The study was approved by the Human Subjects Ethics Sub-Committee of The Hong Kong Polytechnic University (Reference no: HSEARS20190201002-01).

### 2.1. Study Design and Sample

This was a case series study, and participant consent was received prior to the intervention. The study sample consists of twelve individuals with SCI and ten healthy subjects (non-SCI) who were scanned using a 3D ultrasound imaging system [41,42] and had their data evaluated to observe the sagittal spinal profile. In addition, three people with SCI (P10, P11 and P12) with complete tetraplegia participated further to receive the intervention after analyzing the sagittal profile (Table 1).

ASIA has classified SCI as complete/incomplete and graded it into five levels of impairment: ASIA-A: complete injury, no sensory and motor function in the sacral segment (S4–S5); ASIA-B: incomplete injury, sensory but no motor function is preserved below the NLI, while some sensation is preserved in the sacral segment (S4–S5); ASIA-C: incomplete injury, motor function is preserved below the NLI, and more than half of the key muscles have muscle grade <3; ASIA-D: incomplete injury, motor function is preserved below the NLI, and half or more of the key muscles have muscle grade ≥ 3; ASIA-E: normal, all motor and sensory functions are unhindered.

### 2.2. Inclusion and Exclusion Criteria

The healthy participants, similar in age and body mass index (BMI) to those with SCI, were recruited for the purpose of evaluating the sagittal spinal alignment. People with any reported or observed spinal issues were excluded. Those individuals with chronic SCI having stable vital parameters and with no secondary complications were selected for the intervention.

### 2.3. Experimental Protocol and Assessment

The stimulation and training protocols were established based on reference to our previous study [39]. Stimulation electrodes were placed at spinous processes between T11–T12 and L1–L2 with reference electrodes at iliac crest. Two specifically designed constant current stimulators (DS8R, Digitimer, Welwyn Garden City, UK) with the waveform inputs from a function generator (AFG1022, manufactured by Tektronix, Inc., Beaverton, OR, USA) were used for stimulation. A burst of 10 kHz with a frequency of 20–30 Hz and intensity between 95–115 mA was delivered during each session, which lasted for 45–60 min, three sessions per week. The intervention was administered for twelve weeks, and it included TSCS with TSR activities for trunk [39]. The pre- and post-assessment was conducted for participants receiving treatment using 3D ultrasound to evaluate the changes in sagittal profile [41,42]. The International Standards for Neurological Classification of Spinal Cord Injury (ISNCSCI) and Magnetic Resonance Imaging (MRI) taken after the injury were used to identify the neurological level of damage and the completeness of the injury.

### 2.4. Scanning Procedure

**Figure 1:**
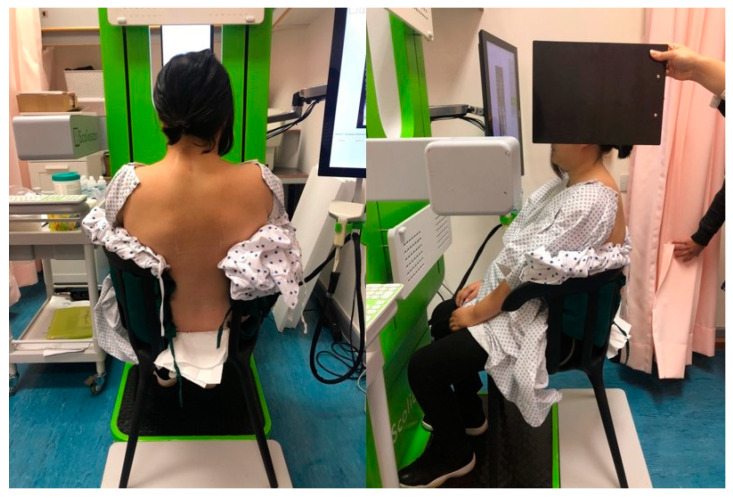
The 3D ultrasound scanning experimental setup and scanning procedure for an individual with SCI in dependent seated position, with both feet placed on the floor, and upper limbs supported over the thighs while maintaining the best upright erect posture.

The posture adopted for non-SCI subjects was independent standing and dependent seated positions (straight or relaxed sitting). The knees were positioned at 90 degrees, and their feet were placed flat on the floor in a neutral position with the upper limbs supported over the thighs when seated. For individuals with SCI, the scanning was performed in dependent seated with their best effort to maintain a straight sitting position. Each scan took around 50–60 s. During the process, no external support was offered, and one operator stood near the person with SCI to prevent him or her from falling. The 3D ultrasound scanning setup is demonstrated in Figure 1.

### 2.5. Data Processing and Analysis

**Figure 2:**
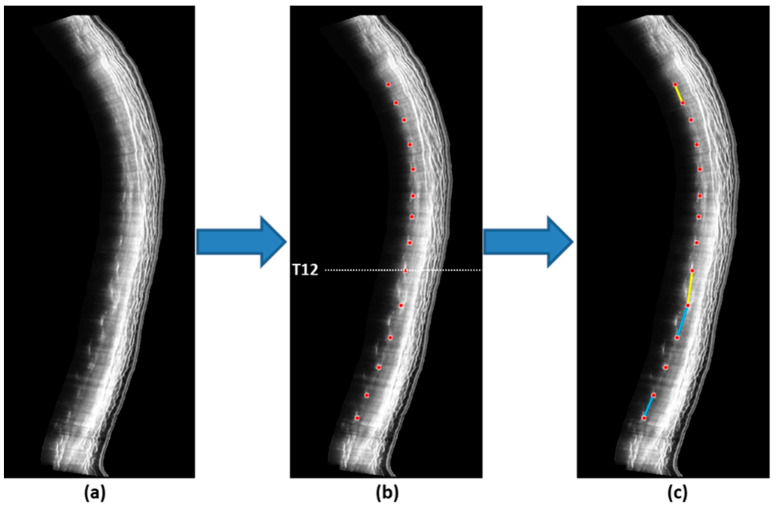
(**a**) Ultrasound sagittal images of the spine; (**b**) Locations of the laminae (red dots) were identified and the corresponding coordinates were extracted for computation of the sagittal curvatures (T12 level is indicated by the white dotted line); (**c**) Thoracic kyphosis was defined as the angle formed between the line T4 and T5 coordinates and the line joining T11 and T12 laminae (yellow); in contrast, lumbar lordosis was defined as the angle formed between the line L1 and L2 coordinates and the line joining L4 and L5 laminae (light blue).

A rater with more than 5 years of experience in studying the human spine using 3D ultrasonography was involved in measuring sagittal curvatures on the ultrasound images. After ultrasound scanning, the ultrasound volume data were then transferred to a customized 3D ultrasound software package (v0.6.4.17, Scoliostudio, Telefield Medical Imaging Limited, Hong Kong) to generate sagittal ultrasound images, which visualize bilateral laminae to evaluate sagittal spinal alignment (Figure 2a). The intra- and inter-rater ICCs for measurement ranging from 0.92 to 0.98 were acquired [43]. Sagittal spinal alignment was assessed in terms of thoracic kyphosis (TK) and lumbar lordosis (LL), using the center of laminae in the sagittal ultrasound images as landmarks (Figure 2b). TK was measured by the averaged intersection angle between the line joining T3 and T4 laminae and the line joining T11 and T12 laminae from the left and right sagittal images; in contrast, LL was measured by the intersection angle between the line joining T12 and L1 laminae and the line joining L4 and L5 laminae from the left and right sagittal images (Figure 2c). All the measurements were conducted using the RadiAnt DICOM Viewer (Medixant, Poznan, Poland) [43,44].

In a nutshell, following manual scanning, the captured ultrasound volume data were transferred to the customized 3D software. The software comprises a number of user interfaces that allow users to view and adjust the positioning and orientation of the original ultrasound B-mode images as well as the projected coronal, sagittal, and transverse views. Operators use the stack of B-mode images and the three projected orthogonal planes in order to produce the best sagittal images. A clear visualization of the laminae, which is required for curvature analysis, characterizes an ideal sagittal image.

### 2.6. Statistical Analysis

Statistical analysis was conducted using SPSS Version 20.0 (IBM, SPSS Inc., Armonk, NY, USA) software. TK and LL acquired from the raters for the individuals with SCI and non-SCI subjects were compared using an independent *t*-test. An ordinary one-way ANOVA was used to evaluate the differences between three postures for non-SCI individuals. Tukey’s multiple comparison test was also used for post-hoc comparison assuming equal variance of the data. In addition, mean ± SD was calculated to show the difference between pre- and post-intervention for people with SCI who received treatment. A *p*-value of 0.05 was set as statistical level of significance.

## 3. Results

### 3.1. Participants’ Characteristics and Overall Sagittal Spinal Profile

As shown in Table 1, the participants were categorized into SCI and non-SCI groups. The SCI group had twelve individuals with chronic SCI (six males and six females) whose age range was 18–57 years (mean age: 28.1 ± 13.3 years). The average BMI was 21.9 ± 2.9 kg/m^2^, and the mean post-SCI duration was 15.9 ± 6.6 years. According to the Neurological Level of Injury and American Spinal Injury Association Impairment Scale (ASIA), the SCI characteristics varied between C6 and T8 injury levels with an incomplete or complete category (ASIA A–D) for people with SCI. The non-SCI group had ten healthy subjects (eight males and two females), and the age range was 16–57 years (mean age: 33.5 ± 11.7 years, mean BMI: 22.6 ± 2.7 kg/m^2^). In addition, regarding the individuals with SCI who participated in treatment, there were three females with an age range of 26–57 years (mean age: 46.0 ± 17.3 years, mean BMI: 21.3 ± 3.1 kg/m^2^), and the mean post-SCI duration was 10.8 ± 8.8 years. Similarly, the level of injury was in the range of C5–C7 with a complete SCI category (ASIA A).

**Figure 3:**
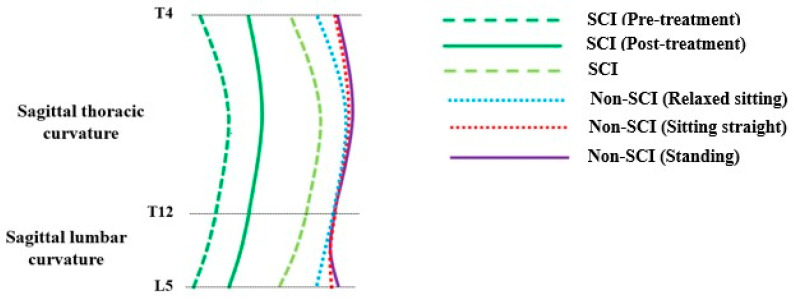
The schematic representation of the sagittal spinal alignment, particularly showing sagittal thoracic curvature and sagittal lumbar curvature of both SCI and non-SCI groups.

The overall sagittal spinal profile was presented for the non-SCI subjects in independent standing and dependent seated positions (straight or relaxed sitting), followed by individuals with SCIs’ profile in a dependent seated posture with their best effort to sit straight. In addition, the pre- and post-sagittal profiles were shown for participants receiving treatment. The sagittal spinal profile appeared similar (C-shaped) for individuals with SCI and non-SCI subjects, when a dependent seated posture was compared to relaxed sitting, but for non-SCI individuals in standing and sitting straight positions, an S-shaped spine was observed. In addition, the progressive change in sagittal thoracic curvature was observed after the treatment compared to the sagittal lumbar curvature. The sagittal spinal curvature for both SCI and non-SCI groups is demonstrated in Figure 3.

### 3.2. Sagittal Curvature Angle Differences for Non-SCI Subjects in Different Postures

**Figure 4:**
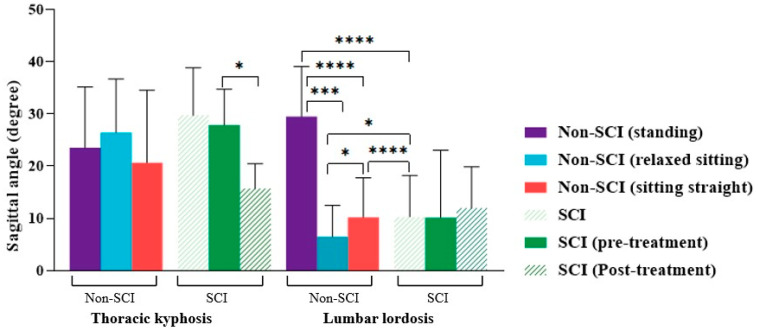
The comparison of the mean sagittal thoracic kyphosis and lumbar lordosis between individuals with SCI and non-SCI subjects (* *p* < 0.05, *** *p* < 0.001, **** *p* < 0.0001).

We found that the mean TK and LL in non-SCI subjects for the standing position was 23.5° ± 11.6° and 29.5° ± 9.6°, respectively. During sitting straight, we observed that TK was 20.3° ± 14.5°, and LL was 10.0° ± 7.9°; in contrast, during relaxed sitting, TK was 26.4° ± 10.3°, and LL was 6.1° ± 9.1°, respectively (Table 2). The ANOVA test showed significant differences for LL (*p* < 0.0001), but no significant difference existed for TK (*p* = 0.5390), for all three postures. Further, the statistical analysis revealed a significant difference between standing and relaxed sitting (*p* = 0.0001), standing and sitting straight (*p* < 0.0001), and sitting straight and relaxed sitting (*p* = 0.0327), respectively, for LL in non-SCI subjects. However, no significant difference was found for TK between all three postures: standing and relaxed sitting (*p* = 0.8569), standing and sitting straight (*p* = 0.8250), and sitting straight and relaxed sitting (*p* = 0.5097), respectively (Figure 4).

### 3.3. Sagittal Curvature Angle Differences between Individuals with SCI and Non-SCI Subjects

The mean TK and LL in individuals with SCI for a dependent seated position was 29.6° ± 9.5° and 7.2° ± 9.7°, respectively (Table 2). In addition, the TK and LL values for people with SCI were greater than non-SCI subjects for: standing (by TK: 6.1° ± 2.1°; LL: 22.3° ± 1.9°), sitting straight (by TK: 10.7° ± 5.0°; LL: 2.8° ± 1.8°), and relaxed sitting (by TK: 3.2° ± 0.8°; LL: 6.6° ± 0.6°), respectively. When TK and LL were compared between individuals with SCI and non-SCI subjects in three different postures (standing, sitting straight, and relaxed sitting). A significant difference was observed for LL between people with SCI and non-SCI subjects for standing (*p* < 0.0001), relaxed sitting (*p* = 0.0146), and sitting straight (*p* < 0.0001) postures, respectively. No significant difference was found for TK between individuals with SCI and non-SCI subjects for all three postures: standing (*p* = 0.1764), relaxed sitting (*p* = 0.4360), and sitting straight (*p* = 0.0771), respectively (Figure 4).

### 3.4. Sagittal Spinal Alignment Changed after Treatment

**Figure 5:**
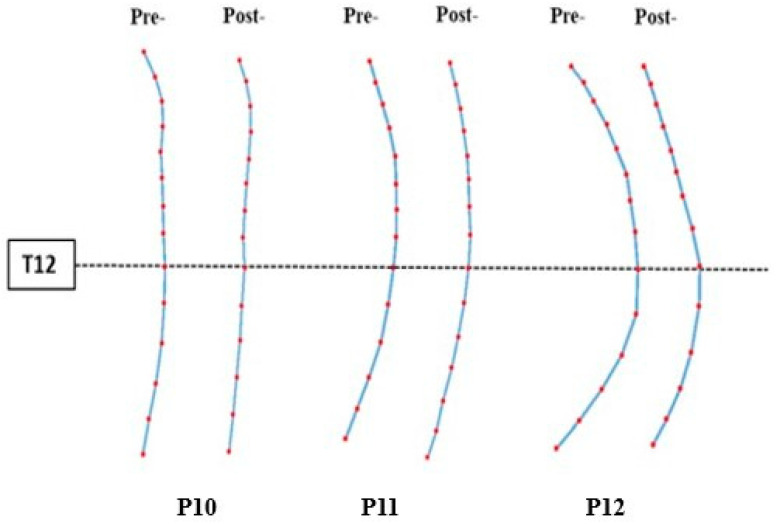
The sagittal profile of the spine based on the coordinates obtained from the sagittal ultrasound images using the laminae landmarks. Red dots indicate the levels of detected vertebral bodies in ultrasound images.

For the participants with SCI who received TSCS intervention, their mean pre-treatment TK and LL angles were 26.6° ± 7.3° and 9.3° ± 13.9°, respectively, whereas post-treatment TK and LL angles were 16.3° ± 5.0° and 11.7° ± 8.3°, respectively. In addition, the TK decreased (from 26.6° to 16.3°), whereas LL increased (from 9.3° to 11.7°) after TSCS treatment. The statistical analysis revealed a significant difference between pre- and post-TK (*p* = 0.0381), but no significant difference was found between pre- and post-LL (*p* = 0.0624). Table 3 demonstrates a decreased mean angle of TK and an increased mean angle of LL. P10 had a reduction in TK by 11.2° (Pre: 27.6°; Post: 16.4°) and an increase in LL by 3.9° (Pre: 0.5°; Post: 4.4°). P11 demonstrated a relatively smaller decrease in TK (Pre: 21.2°; Post: 20.1°) and a slight increase in LL (Pre: 5.1°; Post: 11.2°). P12 had the highest reductions in sagittal angles, where TK (Pre: 34.9°; Post: 10.6°) and LL (Pre: 24.8°; Post: 20.2°) both decreased. It was found that the TK was decreased (by 10.3° ± 2.3°) and LL was increased (by 2.4° ± 5.6°), respectively, after TSCS treatment, resulting a change in sagittal spinal profile. The pre- and post-treatment sagittal spinal profile is demonstrated in Figure 5.

## 4. Discussion

The current study evaluated the differences between sagittal spinal profile in people with chronic SCI and normal individuals in different postures. TSCS treatment was administered on individuals with SCI to determine whether it could restore the normal sagittal curvature. The non-SCI subjects in this study showed a translational increase in TK from sitting straight to standing and from standing to relaxed sitting, whereas LL was significantly reduced when compared between relaxed sitting position with sitting straight and standing postures. One possible reason could be that the lower part of the lumbar spine retains its shape and mobility, while the middle section flattened during the change in posture [45]. A previous study supports this notion, where sagittal alignment changed significantly between a standing and sitting position as assessed in healthy individuals [46]. The changes in sagittal alignment could alter the lumbar spine kinematics, which could affect the load bearing and cause sagittal imbalance [47]. However, an excessive increase in TK (hyperkyphosis) leads to compromised physical function needing clinical attention [18]. It is believed that the change in TK and LL proposes a change in the overall spinal alignment as a self-regulatory mechanism of the body to facilitate stress and load reduction [46]. It is considered that the standing position is the most weight-bearing and optimal posture for the spine [48]. However, for a considerable number of people, such as individuals with SCI, a seated position may be more functional than a standing position. Therefore, the findings could assist clinicians to understand the phenomenon of TK and LL differences in relation to different postures and plan conservative or preventive strategies for maintaining normal spinal alignment.

When healthy subjects moved from standing to sitting, a significant decrease in LL followed by TK was observed. This resulted in a change in the sagittal vertical axis, most likely due to a change in the body’s center of gravity that caused it to migrate forward [46]. The findings in the present study do not support this notion, as TK was significantly greater but LL was lower in people with SCI than in non-SCI individuals for both sitting (straight or relaxed) and standing positions. It is possible that the compensatory posture observed by people with SCI due to trunk instability could have affected their center of gravity. It has been demonstrated that when comparing the spinal alignment of normal individuals in erect sitting to a natural sitting position, the lumbar lordosis has been found to be reduced in erect sitting. Thus, LL loss was even more significant during natural sitting [48]. The present study revealed not just an increase in the degree of TK and LL during a dependent seated position, but also a change in the sagittal profile than other postures for normal individuals. In addition, the sagittal spinal profile appeared similar (C-shaped) between the SCI and non-SCI groups in a dependent seated posture, while it changed when compared to standing or sitting straight. As it is now known that the sagittal profile varies from sitting to standing, some studies showed similar results when assessing spinal curvature in these positions [21,22,49]. In healthy people, a slumped sitting posture and adopting a C-shaped alignment of the spine stretches the posterior ligamentous band and decreases the demand on paraspinal muscles to maintain spinal stability and balance [48]. This function differs in people with SCI, which could be due to trunk muscle paralysis and could be a possible reason for greater TK and a larger C-shaped spine appearance.

The TK in non-SCI individuals in this study for standing and sitting is in the range of 20°–25°, whereas for people with SCI in dependent sitting, it was 30.3°. Those with TK ≥ 30° are predicted to be at risk of chronic pain in the kyphotic region [5]. In the present study, individuals with SCI have shown a TK value higher than this range, indicating they are at risk. The LL showed greater differences between SCI and non-SCI groups than TK for all examined postures. This could be due to a posteriorly tilted pelvis measured till 15° in people with SCI during sitting [50]. Some studies have shown that LL is associated with increased TK [16,51], and the present findings support this statement. Individuals with SCI are at greater risk of developing SCDs that affect their quality of life [52]. The SCDs are found to be influenced by several factors, such as head and sitting position, backrest shape, etc. [53]. Sagittal malalignment has been predicted to be common in people who are mostly dependent on wheel chair due to trunk impairment [14]. In addition, sagittal malalignment may lead to changes in spine kinematics, which can potentially cause low back pain [47], thus affecting mechanical instability and hampering the daily life activities [19]. Meanwhile, there are some controversial views regarding the relationship between the spinal curvature and its function, balance and low back pain [10]. Future studies may consider these factors and investigate whether the spinal profile change could have impact on quality of life.

Currently, surgical management prioritizes sagittal realignment to restore natural spinal curvature in standing posture for normal individuals, since this position is a weight-bearing and the most preferred posture for spine [48], but for a person with SCI, a more functional posture is sitting rather than standing. However, after surgical management of hyperkyphosis, the spine tries to find a new form of equilibrium [54]. In contrast, the current TSCS treatment decreased the TK (Figure 5) and improved the sagittal balance. Furthermore, lateral trunk support used for people with SCI improved the spinal alignment, resulting in a more erect seating posture [14], but this constituted temporary management. In addition, TSCS treatment showed a reversible change in sagittal curvatures. It has been recommended that scoliosis should be examined every 6 months [55], but for individuals with SCI, this recommendation has rarely been followed. Thus, if it could be assessed early, it could be prevented.

The change in sagittal spinal alignment alters tension and flexibility of the spine [56]. Moreover, TSCS to the trunk extensor muscles assists for upright sitting position and could restore a more normal curvature of the low back [32]. A case study demonstrated that an individual with C4 (AIS-A) presented a decrease in thoracic kyphosis from 55° to 34° after the application of invasive epidural stimulation [57]. In contrast, the application of TSCS enhanced erect sitting, thus improving sagittal posture [13]. The current findings support these statements, and the study outcomes may have implications for the management and rehabilitation programs for SCI by identifying the differences in sagittal profile resulting from spinal malalignment. Though the results were promising, which is consistent with an earlier single-subject study [57], it is still difficult to claim effectiveness of TSCS for improving spinal malalignment in people with SCI.

There are several limitations in the current study. The number of participants with SCI receiving TSCS treatment in this study was only three, which was too small to make a conclusive statement. There could be a possibility of potential bias as the evaluator was not blinded in this study. We were unable to evaluate the sagittal alignment for individuals with SCI in a standing position to compare each posture with non-SCI subjects. Although the sagittal spinal curvature in a dependent seated posture appeared identical between people with chronic SCI and non-SCI individuals, conducting the evaluation in the supine position might have increased the results’ reproducibility, despite the fact that the standing position is the standard one in general [47]. Nevertheless, since SCI and non-SCI groups were scanned in different postures, the muscle strength, fatigue and compensatory movement in people with SCI could influence the scanning posture. Therefore, measuring the muscle strength of trunk and spinal muscles could provide more accuracy to the findings. The study findings need to be replicated with a larger sample size, possibly using randomized controlled trials to confirm the effects of TSCS treatment in re-establishing normal sagittal spinal alignment. The design of the back support in a wheelchair could assist in reducing stress on the spine that could lead to spinal deformities [58]. Therefore, further research into studying the sagittal spinal profile in chronic SCI using a manual or motorized wheelchair could help us to understand the differences in sagittal angles, and preventive measures could be considered. Individuals with complete paraplegia have shown greater lordotic deformity with more frequently occurring scoliosis than people with incomplete tetraplegia [59]. In addition, future studies focusing on the level of neurological damage, motor score, severity, and duration of injury are recommended to find out the predictors of SCDs in chronic SCI.

## 5. Conclusions

In the present study, we have demonstrated that the sagittal spinal profile of individuals with chronic SCI differs from that of those without SCI. The people with SCI showed TK values greater than the non-SCI subjects, indicating an increased risk for spinal deformity. Therefore, the correction of the deformity appears to be essential. In addition, after introducing TSCS treatment, the progressive change in sagittal thoracic curvature was observed, indicating a reversible change in sagittal spinal profile. It is believed that this treatment can produce a sustained effect after the period of intervention. Therefore, it is suggested that future studies can include multiple follow-up assessments after the intervention to investigate the sustainability of the treatment effects. Furthermore, the findings may be considered preliminary, and future randomized controlled trials with a larger sample size should be conducted to evaluate the feasibility of this intervention, thus possibly providing convincing proof of its effectiveness.

## Figures and Tables

**Table 1:** SCI and non-SCI participant characteristics.

	Participants	Gender	Age (yr)	BMI (kg/m^2^)	ASIA	NLI	Post-SCI (yr)
	SCI group						
	P1	M	44	18.2	C	C8	20
	P2	F	39	24.8	B	C7	13
	P3	M	36	20.2	B	T1	11
	P4	M	58	20.8	C	C6	18
	P5	M	31	26.7	B	C7	12
	P6	F	48	18.0	C	T6	22
	P7	M	50	23.0	B	T2	13
	P8	F	61	25.0	C	C8	26
	P9	M	38	22.9	D	T8	23
			28.1 (Mean) 13.3 (SD)	21.9 (Mean) 2.9 (SD)			15.9 (Mean) 6.6 (SD)
Received TSCS	P10	F	57	24.8	A	C6	2
	P11	F	55	18.8	A	C7	19
	P12	F	26	20.4	A	C5	12
			46.0 (Mean) 17.3 (SD)	21.3 (Mean) 3.1 (SD)			10.8 (Mean) 8.8 (SD)
	Non-SCI group						
	S1	M	28	20.2	-	-	-
	S2	M	57	19.7	-	-	-
	S3	F	47	26.6	-	-	-
	S4	F	40	22.0	-	-	-
	S5	M	16	19.5	-	-	-
	S6	M	25	24.7	-	-	-
	S7	M	32	20.4	-	-	-
	S8	M	28	25.0	-	-	-
	S9	M	29	25.9	-	-	-
	S10	M	33	22.6	-	-	-
			33.5 (Mean) 11.7 (SD)	22.6 (Mean) 2.7 (SD)			

BMI: Body Mass Index; ASIA: American Spinal Injury Association Impairment Scale; NLI: Neurological Level of Injury; F: Female; M: Male; SD: Standard Deviation.

**Table 2:** Sagittal curvature angle for SCI and non-SCI groups.

Sagittal Angle (Degree)	SCI	Non-SCI		
		Standing	Sitting Straight	Relaxed Sitting
TK (mean ± SD)	29.6 ± 9.5	23.5 ± 11.6	20.3 ± 14.5	26.4 ± 10.3
LL (mean ± SD)	7.2 ± 9.7	29.5 ± 9.6	10.0 ± 7.9	6.1 ± 9.1

Thoracic kyphosis: TK; lumbar lordosis: LL; SD: standard deviation.

**Table 3:** Sagittal curvature angle pre and post treatment.

P10 (Mean in Degree)		P11 (Mean in Degree)		P12 (Mean in Degree)		Average (Mean ± SD)	
Pre	Post	Pre	Post	Pre	Post	Pre	Post
(TK) 27.6	16.4	21.2	20.1	34.9	10.6	26.6 ± 7.3	16.3 ± 5.0
(LL) 0.5	4.4	5.1	11.2	24.8	20.2	9.3 ± 13.9	11.7 ± 8.3

Thoracic kyphosis: TK; lumbar lordosis: LL; SD: standard deviation.

## Data Availability

The data generated from this work can be obtained from the corresponding author upon reasonable request.
